# Vasopressor Effect of Indigo Carmine in the Management of Refractory Neurogenic Shock

**DOI:** 10.7759/cureus.41004

**Published:** 2023-06-26

**Authors:** Natsuko Kurozumi, Nobuko Fujita, Takuto Kaneko, Maiko Shinoda, Seiki Abe

**Affiliations:** 1 Anesthesiology, St. Luke’s International Hospital, Tokyo, JPN

**Keywords:** vasopressor, neurogenic shock, indigo carmine, catecholamine-refractory shock, cervical cord injury

## Abstract

Neurogenic shock in patients with spinal cord injuries can be fatal. Catecholamines are commonly used for the management of neurogenic shock; however, the treatment of catecholamine-refractory neurogenic shock remains challenging. A 78-year-old woman with neurogenic shock from cervical cord injury underwent posterior cervical spine decompression and fixation. The patient’s blood pressure could not be maintained with catecholamine administration throughout the surgery. Therefore, indigo carmine was administered, and an effective increase in blood pressure was achieved. The postoperative course was uneventful. The findings from this case indicate that indigo carmine may have an effective vasoconstrictive action in patients with neurogenic shock who do not respond to catecholamines.

## Introduction

Patients with spinal cord injuries exhibit hypotension due to disorders of the sympathetic nervous system. Dysfunction of the sympathetic nerves, which pass through the lateral horn of the spinal cord, leads to sympathetic hypoactivity. Severe spinal cord injury occurring above the thoracic T6 vertebrae can lead to the loss of supraspinal sympathetic control of cardiovascular functions [1,2]. Neurogenic shock can be fatal [3], and maintenance of circulatory control is important for the prevention of spinal ischemia and secondary insult, even in the absence of shock [4]. The management of blood pressure (BP) may require the administration of intravenous fluid and blood products, vasopressors, and inotropes [5]. Catecholamines are commonly used as vasopressors to manage shock; nevertheless, managing catecholamine-refractory cases remains challenging.

Herein, we describe a case of an elderly woman with catecholamine-refractory neurogenic shock whose BP was effectively increased via the administration of indigo carmine, a dye clinically used for diagnostic purposes and organ identification.

## Case presentation

A 78-year-old woman (height, 154 cm; weight, 51 kg) developed weakness in both lower limbs after falling and was transported to our hospital. The patient presented with cervical cord injury and neurogenic shock and underwent immediate decompression and cervical spine corrective surgery. She had a history of hypertension and dyslipidemia and performed her daily activities independently.

**Figure 1:**
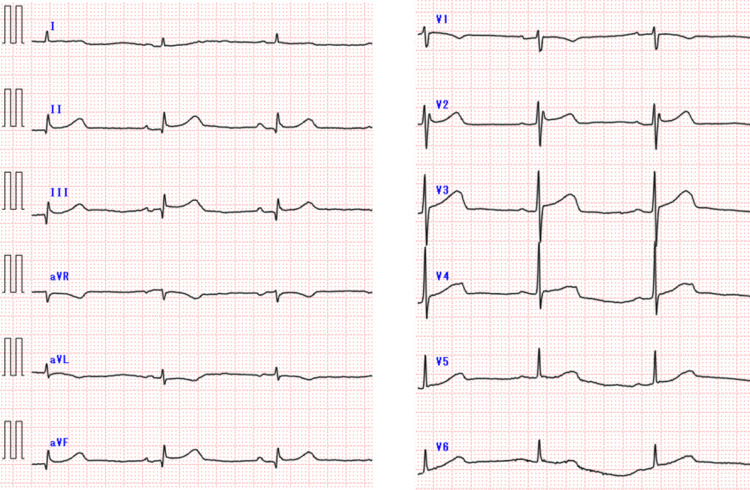
12-lead electrocardiogram A 12-lead electrocardiogram showing sinus bradycardia (heart rate [HR]: 49 bpm).

Her vital signs on admission were as follows: Glasgow coma scale score (eye-opening/best verbal response/best motor response), E3V4M5; BP, 80/40 mmHg; heart rate (HR), 49 bpm; respiratory rate, 18 breaths/min; percutaneous oxygen saturation, 96% (room air); and body temperature, 36.1°C. Weakness in both lower limbs, difficulty in flexion and extension of both hands and fingers, and sensory disturbances below L2 were observed. Blood gas analysis revealed that respiratory function was stable (partial pressure of arterial oxygen, 71.2 mmHg; partial pressure of arterial carbon dioxide, 44.5 mmHg). Laboratory tests revealed mild anemia (hemoglobin, 10.5 g/dL) and no elevation of the myocardial enzyme levels (creatine kinase-muscle brain type, 24 U/L; troponin T, 0.038 ng/dL). A 12-lead electrocardiogram revealed sinus bradycardia (HR, 49 bpm; Figure 1), whereas transthoracic echocardiography revealed normal heart movement and no abnormal cardiac structure.

**Figure 2:**
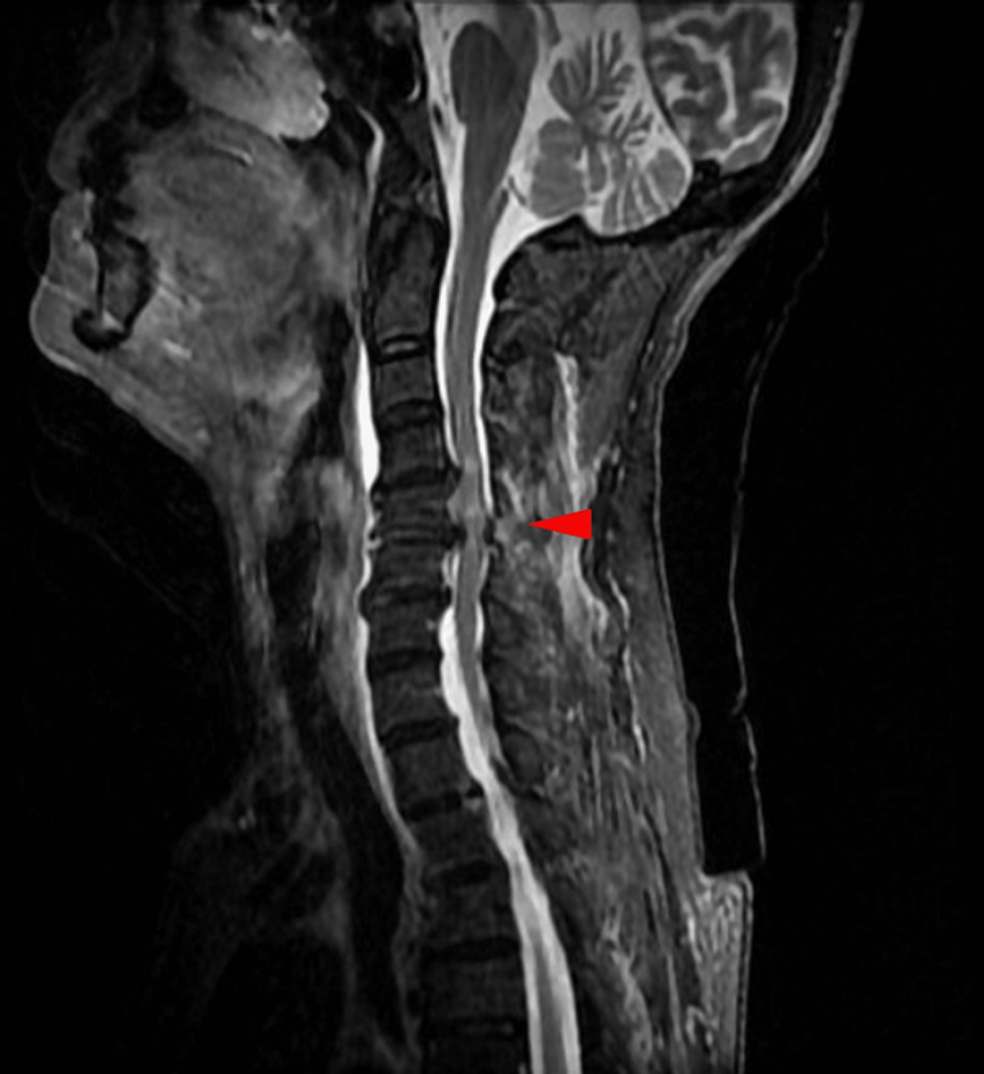
Cervical magnetic resonance imaging The image shows spinal cord injury and spinal stenosis at C4/C5 and C5/C6 (arrowhead).

Cervical magnetic resonance imaging revealed spinal cord injury, spinal stenosis at C4/C5 and C5/C6, and anterior spinal hematomas from C2 to thoracic T3 (Figure 2). A computed tomography scan of the head was normal, and no fractures or hemorrhages were observed. No other major injuries were detected.

**Figure 3:**
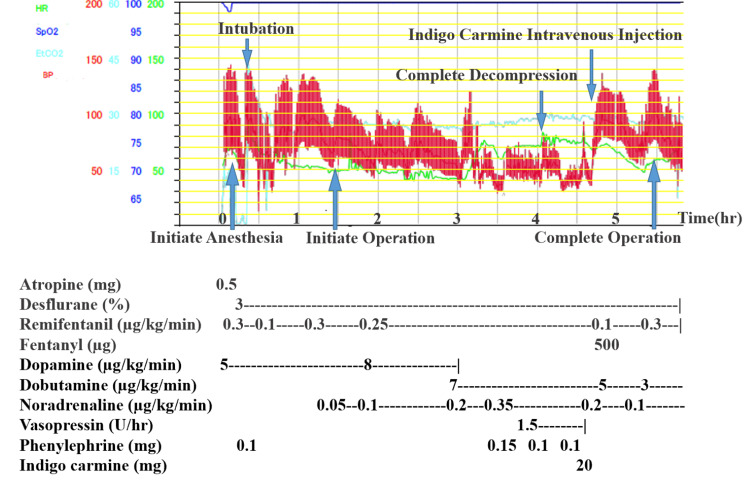
Anesthesia course Circulatory dynamics were unstable even with adequate catecholamine administration. However, the blood pressure was elevated immediately after administration of indigo carmine.

Figure 3 shows the anesthesia course. Bradycardia (HR, 30 bpm) was observed on arrival in the operation room despite the administration of 5 μg/kg/min of dopamine, and hemodynamic stability was achieved temporarily by the administration of 0.5 mg of atropine. Following the induction of anesthesia with 3.0 mg of midazolam and 0.3 μg/kg/min of remifentanil, the systolic BP decreased to 70 mmHg. The patient was intubated with a fiber-optic bronchoscope. The systolic BP was raised via administering 0.1 mg of phenylephrine and stimulation of intubation. Subsequently, inhalation anesthesia with desflurane was used for maintenance. The administration of noradrenaline was initiated at the same time as initiating the operation and gradually increased to maintain the BP. During the operation, the HR was maintained at 40-50 bpm, with a systolic BP of <90 mmHg. Although the dopamine dose was increased to 8 μg/kg/min, there was no improvement in bradycardia or hypotension. Therefore, dopamine was switched to dobutamine to increase cardiac output. The switch resulted in an increase in the HR to 70 bpm; however, the BP dropped to approximately 70 mmHg even after administering 7 μg/kg/min of dobutamine, 0.35 μg/kg/min of noradrenaline, and 1.5 U/h of vasopressin. As even noradrenaline at a dose of 0.35 μg/kg/min could not elevate the BP, the administration of vasopressin was initiated. Physical findings, blood gas analysis, and electrocardiogram ruled out other causes of shock, such as anaphylaxis, bleeding, and cardiogenicity. Nevertheless, hemodynamic stability could not be achieved even after decompression of the spinal cord was completed, with the systolic BP dropping to 60 mmHg. Thus, 20 mg of indigo carmine was administered intravenously, following which the systolic BP immediately increased to 130 mmHg. The patient’s BP stabilized, and the vasopressor doses were gradually reduced to 3 μg/kg/min of dobutamine and 0.1 μg/kg/min of noradrenaline.

The postoperative course was uneventful. Catecholamines were withdrawn on postoperative day (POD) 9, and the patient was transferred to a rehabilitation hospital on POD 34.

## Discussion

Severe spinal cord injury occurring above thoracic T6 results in poor supraspinal control of the sympathetic nervous system, and parasympathetic cardiac responses via the vagus nerve are the only supraspinal control of the heart, leading to cardiovascular dysfunction [1,2]. Neurogenic shock occurs more commonly in patients with a complete cervical spine injury, and those with a higher level injury in the cervical spine have a significantly greater requirement for a cardiovascular intervention [6]. In addition, pulmonary complications, gastrointestinal complications, electrolyte disorders, and urologic complications may occur. Neurogenic shock occurs in approximately 20% of patients with spinal cord injury above thoracic T6 [7]. An average systolic BP of >80 mmHg should be maintained to prevent spinal ischemia in patients with spinal cord injury [4]. Fluid and blood transfusions or vasopressors, such as catecholamines, are often administered to overcome a hypotensive state. However, excessive fluid and blood transfusion may increase spinal cord swelling; thus, transfusions should be carefully regulated [8]. Some patients with spinal cord injury present with catecholamine-refractory neurogenic shock as observed in the present case. Epinephrine may be administered to improve hypotension; however, it has been reported that an increase in cellular metabolism, which is an effect of epinephrine, worsens the patient’s prognosis [9] and should be avoided. In the present case, an increase in BP was observed following the administration of indigo carmine, which helped manage the shock without the administration of epinephrine.

**Figure 4:**
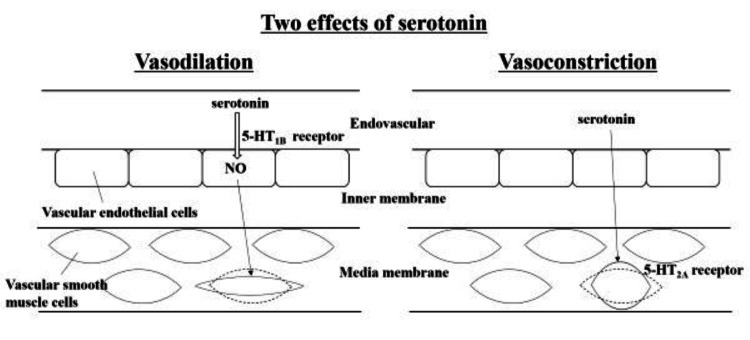
The vasoconstrictor and vasodilator effects of serotonin Serotonin acts on the vascular smooth muscle cells and shows a vasopressor effect that allows vascular smooth muscles to contract. It also acts on the vascular endothelial cells and produces nitric oxide, having a vasodilator effect that relaxes vascular smooth muscles.

Indigo carmine is a dye that is clinically used for organ identification in urological and mammary gland surgeries; however, it is known to often increase BP [10]. To our knowledge, there are no reports on its use for the management of neurogenic shock. Indigo carmine affects circulatory dynamics as its chemical structure is similar to that of serotonin [10,11], which has a vasopressor effect. Serotonin acts directly on the 5-HT2A receptors on the vascular smooth muscle cells, showing a vasopressor effect and causing vascular smooth muscles to contract. Additionally, serotonin also acts on the 5-HT1B receptors of the vascular endothelial cells and produces nitric oxide, exerting a vasodilator effect that relaxes the vascular smooth muscles (Figure 4) [12]. Depending on the situation, indigo carmine can show vasoconstrictor or vasodilator effects. The drug reportedly exhibits a vasodilator effect when the sympathetic tone is dominant, as observed under adrenaline administration, whereas it shows a vasoconstrictor effect when the parasympathetic tone is dominant, as observed during general anesthesia or subarachnoid/epidural anesthesia of the spinal cord [11,13]. When indigo carmine was injected into 20 patients undergoing urological surgery under general anesthesia or spinal subarachnoid anesthesia, the systolic and diastolic BP increased by 25 and 14 mmHg, respectively [11]. Our patient had a spinal cord injury and underwent surgery under general anesthesia; this can imply parasympathetic dominance. We speculated that indigo carmine would exhibit a vasopressor effect in this case and observed BP elevation with the medication.

We surmised that the patient’s BP was increased by the administration of indigo carmine, rather than by decompression of the spinal cord, for the following reasons: (1) the BP was elevated immediately after the administration of indigo carmine, and (2) it has been reported that patients with cervical cord injury can have prolonged hypotension till the repair of the spinal cord damage. It has been reported that neurogenic shock may persist from 30 minutes to five weeks, depending on the individual [14]. In this case, we considered that indigo carmine was effective in maintaining the BP until neurogenic shock was improved after decompression. A previous study reported that the elevation in the BP caused by the administration of indigo carmine returned to normal in 5-35 minutes (22 minutes on average) [11]. The patient’s BP stabilized after administering indigo carmine, and we were able to reduce the dose of the vasopressor. Nevertheless, the effects of indigo carmine on circulatory dynamics remain unclear. Further cases are required to evaluate its effect.

## Conclusions

We experienced a rare case of severe neurogenic shock that could not be managed even with the administration of catecholamines, which would have a poor prognosis. Indigo carmine is commonly used as a dye but often results in an unexpected increase in BP. The chemical structure of indigo carmine is similar to that of serotonin, which could explain the increase in the BP in this case. We successfully managed catecholamine-refractory neurogenic shock using indigo carmine. Thus, indigo carmine can be considered as an option to raise BP in patients with spinal cord injuries. The effects of indigo carmine on circulatory dynamics are unclear; therefore, further cases are required to evaluate its effect and prove its usefulness in spinal cord injuries.
